# An HIV-Positive Patient With Disseminated Histoplasmosis Complicated by Histoplasma Ileitis-Induced Small Bowel Obstruction

**DOI:** 10.7759/cureus.14677

**Published:** 2021-04-25

**Authors:** Christina Lee, Jake Jasurda, Alison Wing

**Affiliations:** 1 Internal Medicine, University of Minnesota Medical School, Minneapolis, USA; 2 Internal Medicine, Regions Hospital, St. Paul, USA

**Keywords:** disseminated histoplasmosis, gastrointestinal histoplasmosis, histoplasma enteritis, small bowel obstruction, hiv aids, cmv duodenitis

## Abstract

A 43-year-old male has a medical history of Human immunodeficiency virus (HIV) with no anti-retroviral therapy for six years prior to admission. He presented from an outside hospital with 40 lbs weight loss over one year, worsening abdominal pain, and odynophagia, with CT-confirmed small bowel obstruction (SBO) in the setting of untreated cytomegalovirus (CMV) ileitis. Treatment for both the untreated HIV and CMV ileitis was started during this hospitalization, and his hospital course was complicated by disseminated histoplasmosis in his lungs and GI tract, leading to stricture and a recurrent SBO. This case report will focus on an unusual complication of untreated HIV and a late diagnosis of histoplasmosis: Histoplasma ileitis-induced stricture and recurrent SBO. To date, there are a limited number of reports that describe gastrointestinal histoplasmosis in HIV patients, and SBO remains a rare and serious complication of disseminated histoplasmosis.

## Introduction

*Histoplasma capsulatum* is endemic to North America, Central America, and many countries of South America and also occurs in China, India, Southeast Asia, Africa, Australia, and Europe [1]. *Histoplasma capsulatum* is a dimorphic fungus that primarily presents as a self-limited respiratory illness in immunocompetent individuals [2]. In patients with an intact cell-mediated immune response, *Histoplasma capsulatum* is actively cleared by T-cell activated macrophages, which engulf the organism and clear the yeast cells, leaving the patients asymptomatic [3]. However, immunocompromised patients, such as those infected with HIV, can present with disseminated and life-threatening infections [3]. Due to the lack of macrophage activation from helper T cells, the yeast cells remain engorged within the macrophages and spread through the lymphatics system [2-3]. Thus, the most common extrapulmonary sites involved include the lymph nodes, liver, spleen, and bone marrow [3]. In a retrospective review of 18 HIV-seropositive patients with confirmed gastrointestinal histoplasmosis, only three patients’ hospital courses were complicated with high-grade small bowel obstruction (SBO) [3], and in another retrospective study of 23 HIV patients with confirmed gastrointestinal histoplasmosis, only three patients had SBO complications [4]. This data demonstrates the rarity of this complication.

We present an unusual case of a 43-year-old male with untreated HIV, who was diagnosed with histoplasmosis in his terminal ileum, which leads to a rare complication of recurrent high-grade SBO.

## Case presentation

A 43-year-old male from Guatemala presented as a direct admission for untreated HIV complicated by cytomegalovirus (CMV) ileitis, 40 lb weight loss over one year, worsening abdominal pain, and odynophagia. His medical history was notable for untreated HIV diagnosed six years prior to admission and a remote history of methamphetamine abuse.

Two months prior to his hospitalization, the patient was hospitalized at an outside hospital in California for abdominal pain and a 40 lb weight loss. The initial CT abdomen showed an SBO and thickening in the terminal ileum, concerning inflammatory bowel disease (IBD). An esophagogastroduodenoscopy (EGD) at that time was unremarkable though biopsies were obtained. A colonoscopy demonstrated ulcers in the terminal ileum, and the patient was ultimately discharged on a two-week course of prednisone 40 mg with concern for new onset of Crohn’s disease. The biopsy pathologies subsequently returned with intraepithelial lymphocytosis on the duodenal tissue biopsy, scattered CMV inclusions by immunochemistry on the terminal ileum tissue biopsy, and negative for IBD, but these pathology reports, unfortunately, resulted after the patient had been discharged and he was unable to be reached to initiate therapy. There were no stains performed for histoplasmosis specifically, although the reasons are unclear.

Two months later, he was again admitted to the hospital for worsening abdominal pain. Upon admission, the patient stated that he had been having diffuse abdominal pain and intermittent non-bloody diarrhea for two months, as well as weight loss of 40 lbs over the past year. He had also experienced persistent throat pain with swallowing for the past month and had been eating significantly less due to the odynophagia. He denied dysphagia to solids and liquids, acid reflux, fever, chills, headache, joint pains, and new rashes. He stated that he had not taken any anti-HIV medications since diagnosis six years ago.

On initial physical examination, vital signs were as follows: afebrile, blood pressure (BP) 114/86, heart rate (HR) 99, respiratory rate (RR) 18, SpO2 100% on RA, and BMI 26. The physical exam was unremarkable.

The patient’s labs were notable for the following: Creatinine (Cr) 0.63 mg/dL, white blood cells (WBC) 3.8 10e9/L, Hgb 10 g/dL, MCV 77.5 fl, Platelet 341 10e9/L, absolute lymphocyte 0.7 10e9/L, CD4 47, HIV viral load 150,000, alkaline phosphatase 279 IU/L, AST 67 U/L.

He stopped smoking 30+ years ago, denied alcohol use, and current use of methamphetamine, and stated that he was currently only sexually active with his wife. He moved to the United States from Guatemala 22 years ago. He lived in California for 15 years and then lived in Minnesota for the past seven years, and has been living at his friend’s home in the suburbs of Minnesota. Due to his undocumented status, he has never worked. He denied any known outdoor exposures to Histoplasma in Guatemala and it is unclear if he has had exposures to *Histoplasma* since moving to the United States.

Due to his ongoing odynophagia, an EGD was again performed with a biopsy obtained that showed non-specific reactive changes in the squamous mucosa of the esophagus and mildly increased intraepithelial lymphocytes with normal villous architecture in the duodenum. Ganciclovir was started for untreated CMV ileitis.

**Figure 1:**
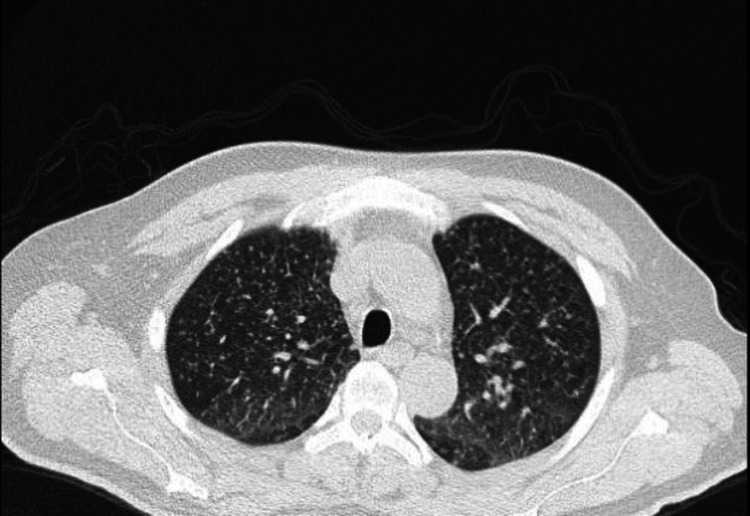
CT chest with contrast demonstrating extensive random miliary nodules bilaterally with an upper lobe predominance.

A chest X-ray was obtained for a cough, and it demonstrated a diffuse miliary pattern, with subsequent CT chest demonstrating extensive miliary nodules bilaterally with an upper lobe predominance (Figure 1). Subsequent urine Histoplasma antigen was positive. Also, after three days post collection, Histoplasma grew from the right upper lung lobe bronchoalveolar lavage (BAL) fungal culture. Fungal elements consistent with *Histoplasma capsulatum,* per pathology, were found on microscopy, and identification was also confirmed with polymerase chain reaction (PCR) test. Aerobic and anaerobic blood cultures were negative, and no fungal blood cultures were obtained.

**Figure 2:**
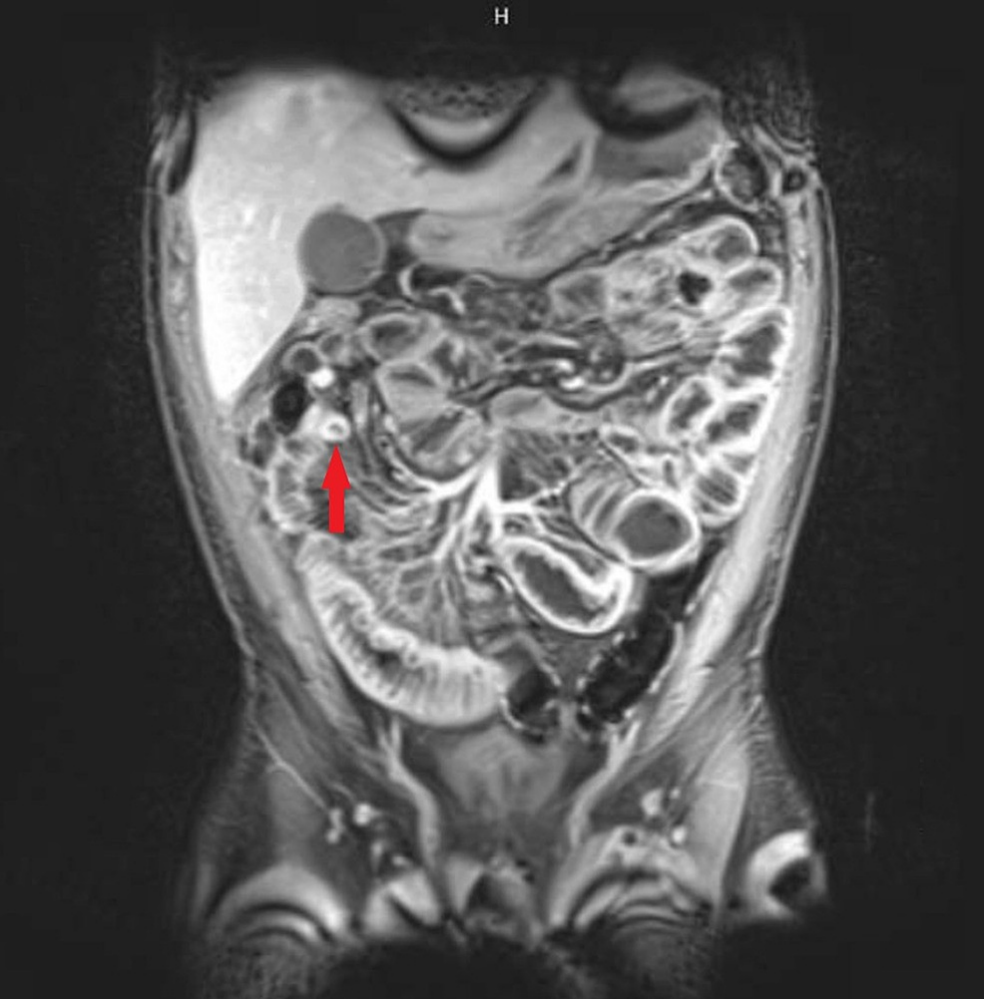
Magnetic resonance enterography abdomen/pelvis showing evidence of active enteritis and stricture in the terminal ileum (red arrow).

**Figure 3:**
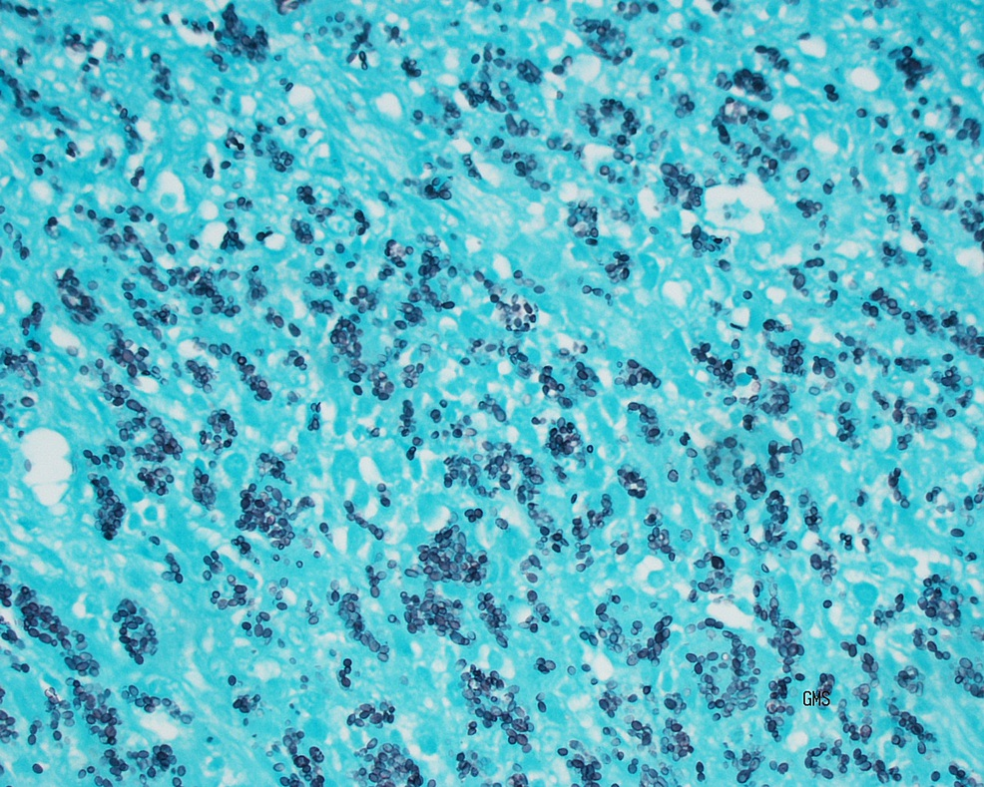
Grocott-Gomori’s methenamine silver (GMS) stain of the biopsy from the terminal ileum stricture site demonstrating Histoplasma capsulatum.

Seven days after admission, the patient developed worsening abdominal pain and a CT abdomen/pelvis at that time demonstrated markedly dilated distended loops of small bowel with decompressed loops of ileum and colon consistent with a high-grade SBO. Subsequent magnetic resonance (MR) enterography abdomen/pelvis showed evidence of long segment active enteritis involving much of the distal ileum with narrowing in the distal ileum in the right mid-abdomen suspicious for a stricture and ongoing colitis in the ascending colon (Figure 2). The patient underwent a laparoscopic-assisted ileocecectomy and ileocolic anastomosis, and tissue biopsy demonstrated histoplasmosis-associated enteritis in the terminal ileum (Figure 3). The Grocott-Gomori’s methenamine silver (GMS) stain of the terminal ileum highlighted abundant fungal organisms, morphologically consistent with *Histoplasma capsulatum*. CMV stain of the colon (terminal ileum, cecum, appendix) was negative for CMV inclusions.

The patient was ultimately started on antiretroviral therapy (bictegravir, emtricitabine, and tenofovir alafenamide) for his previously untreated HIV. For his disseminated histoplasmosis, the patient was treated with amphotericin B intravenously (IV) for four consecutive weeks and was transitioned to voriconazole, rather than itraconazole with the patient’s transaminitis, for 12 months.

Prior to discharge, the patient decided to move back to his home state. The infectious disease (ID) physicians contacted the patient’s home primary care physician and his ID team prior to discharge for continuity of care. Due to the patient’s move outside of our healthcare system, no follow-up about his treatment progress is available.

## Discussion

Diagnosis of disseminated histoplasmosis can be delayed since symptomatology is highly variable and non-specific [5]. The most common presenting symptoms are fever, hepatosplenomegaly, weight loss, and hematologic findings, with GI symptoms occurring in approximately 33% of patients [5].

Lesions within the GI tract can present with polyploid masses and/or ulcerations, most commonly presenting in the ileum and colon, and due to these characteristics, false diagnoses of Crohn’s disease or ulcerative colitis could be made [2]. For instance, in our patient’s case, although it is unclear why *Histoplasma* was not detected during the first biopsy at the outside hospital, it can be speculated that GI histoplasmosis was not on the differential since there was high suspicion for IBD, and thus the tissue was not stained specifically for* Histoplasma capsulatum*. Another possibility is that the random sample did not have any *Histoplasma capsulatum* present.

However, initiation of immunosuppressive therapy such as steroids or TNF-alpha inhibitors for presumed IBD can lead to further progression of histoplasmosis [6]. Thus, primarily in immunocompromised patients, for instance with HIV/AIDS, if there is a high degree of clinical suspicion for Histoplasma involvement in the GI tract due to symptoms such as, but not limited to, weight loss, GI bleeding, perforation, and/or obstructions, clinicians should assess for the possible diagnosis of GI histoplasmosis [4]. Specifically, in a case such as that of our patient, with untreated HIV, weight loss, and recurrent SBO, it was important to include disseminated histoplasmosis with GI involvement in the diagnostic differential.

Disseminated histoplasmosis and specifically, GI histoplasmosis should be suspected at a higher rate in HIV-seropositive patients, who have not received active antiretroviral therapy [3]. The definite diagnosis of histoplasmosis is made showing fungi on histopathology, cytopathology, or cultures, with granulomas, caseating or non-caseating [1]. With GMS or hematoxylin-eosin staining, narrow-based budding yeasts can be seen within tissues or engulfed by macrophages [1]. A case series showed fungal organisms on biopsy specimens in 95% of cases and on the culture of GI lesions in 88% of cases [3]. In comparing antigen testing, a study concluded that a single antigen test, such as using Histoplasma urinary antigen alone, sufficed as an initial screen for suspected histoplasmosis [7]. However, a study found BAL* Histoplasma* antigen to have higher sensitivity than both the serum and urine antigen and was the only evidence of antigen positivity in 10% of the patient samples [8-9]. Antibodies are typically produced four to six weeks after the exposure and may persist for years, limiting their usefulness in the diagnosis of acute histoplasmosis. Molecular diagnostics, specifically PCR does not have high sensitivity as a diagnostic tool [1].

If there is high suspicion for disseminated histoplasmosis, the highest diagnostic sensitivity is obtained by using multiple diagnostic tests, including cytopathology and/or histopathology, culture, both urine and serum antigen detection, and antibody detection [1]. Overall, early suspicion and diagnosis of disseminated histoplasmosis, followed by prompt treatment are necessary, since mortality can be as high as 80% [10].

In two separate retrospective studies with HIV-seropositive patients with confirmed GI histoplasmosis [2-3], SBO was a rare complication. Although SBOs are rare complications in disseminated histoplasmosis, it is important that clinicians stay vigilant in assessing worsening abdominal pain and the need for repeat imaging, because some patients may, as in our patient’s case, require and benefit from surgical interventions.

## Conclusions

In immunocompromised patients, it is important for clinicians to have a high degree of suspicion for Histoplasma involvement in the GI tract, especially in cases already identified as disseminated histoplasmosis. Weight needs to be placed on obtaining biopsies and cultures during procedures, like bronchoscopy, endoscopy, and surgical interventions to confirm the diagnosis, especially since mortality of untreated disseminated histoplasmosis can be very high. Endemic mycosis can present in many unusual ways in someone with confirmed and untreated HIV, like in our patient’s case, and epidemiological risk is also very important to take into consideration. We believe that, with earlier suspicion, patients will receive prompt and needed antifungal treatment, leading to better overall outcomes for patients.
